# Food Additives and Hyperactivity

**DOI:** 10.1289/ehp.11182

**Published:** 2008-06

**Authors:** Bernard Weiss

**Affiliations:** Department of Environmental Medicine, University of Rochester School of Medicine and Dentistry, Rochester, New York, E-mail: bernard_weiss@urmc.rochester.edu

In the December 2007 Forum article on the links between food additives and hyperactivity, Barrett (2007) offered a somewhat distorted perspective on the public health implications of these additives. Barrett described a clinical trial testing the proposition that consumption of a blend of artificial food flavors and sodium benzoate induces changes in children’s behavior (McCann et al. 2007). The results of that study support such a claim.

Barrett (2007) fumbled the significance of the trial (McCann et al. 2007) for environmental health. The Forum article emphasized how food additives might contribute to the clinical diagnosis of attention deficit/hyperactivity disorder rather than on the more significant finding that food additives, particularly synthetic colors at levels prevailing in the diet, induce adverse behavioral responses. This is hardly a novel finding. In 1980, such effects were documented in two different groups of subjects with two different experimental designs (Swanson and Kinsbourne 1980; Weiss et al. 1980). Many later publications have confirmed their results. I briefly reviewed the data in *Environmental Health Perspectives* (Weiss 2000).

According to Barrett (2007), a Food and Drug Administration (FDA) official, Mike Herndon, maintains that the agency sees “… no reason at this time to change our conclusions that the ingredients that were tested in this study that currently are permitted for food use in the United States are safe for the general population.” This is a rather baffling statement. In fact, our study (Weiss et al. 1980) was funded by the FDA, and its results, along with a number of others from that period, definitively demonstrated adverse behavioral effects of synthetic food colors (Weiss 1982). During the intervening years, with a plethora of confirmations, the FDA has remained blindly obstinate. It continues to shield food additives from testing for neurotoxicity and apparently believes that adverse behavioral responses are not an expression of toxicity.

Herndon and the FDA should seriously consider what the late Philip Handler said about balancing risks and benefits:

A sensible guide would surely be to reduce exposure to hazard whenever possible, to accept substantial hazard only for great benefit, minor hazard for modest benefit, and no hazard at all when the benefit seems relatively trivial. (Handler 1979)

The FDA has never clarified the health benefits of artificial food colors.
